# Gender Bias in Human Systemic Lupus Erythematosus: A Problem of Steroid Receptor Action?

**DOI:** 10.3389/fimmu.2018.00611

**Published:** 2018-03-28

**Authors:** Virginia Rider, Nabih I. Abdou, Bruce F. Kimler, Nanyan Lu, Susan Brown, Brooke L. Fridley

**Affiliations:** ^1^Department of Biology, Pittsburg State University, Pittsburg, KS, United States; ^2^Center for Rheumatic Diseases, St. Luke’s Hospital, Kansas City, MO, United States; ^3^Department of Radiation Oncology, University of Kansas Medical Center, Kansas City, MO, United States; ^4^Division of Biology, Kansas State University, Manhattan, KS, United States; ^5^Department of Biostatistics, University of Kansas Medical Center, Kansas City, MO, United States

**Keywords:** systemic lupus erythematosus, human T cells, estradiol, estrogen receptors, glucocorticoid receptors

## Abstract

Systemic lupus erythematosus (SLE) is a chronic systemic autoimmune disease resulting from abnormal interactions between T and B cells. The acquisition of SLE is linked to genetic susceptibility, and diverse environmental agents can trigger disease onset in genetically susceptible individuals. However, the strongest risk factor for developing SLE is being female (9:1 female to male ratio). The female sex steroid, estradiol, working through its receptors, contributes to the gender bias in SLE although the mechanisms remain enigmatic. In a small clinical trial, monthly administration of the estrogen receptor (ERα) antagonist, ICI182,780 (fulvestrant), significantly reduced disease indicators in SLE patients. In order to identify changes that could account for improved disease status, the present study utilized fulvestrant (Faslodex) to block ERα action in cultured SLE T cells that were purified from blood samples collected from SLE patients (*n* = 18, median age 42 years) and healthy control females (*n* = 25, median age 46 years). The effects of ERα antagonism on estradiol-dependent gene expression and canonical signaling pathways were analyzed. Pathways that were significantly altered by addition of Faslodex included T helper (Th) cell differentiation, steroid receptor signaling [glucocorticoid receptor (GR), ESR1 (ERα)], ubiquitination, and sumoylation pathways. ERα protein expression was significantly lower (*p* < 0.018) in freshly isolated, resting SLE T cells suggesting ERα turnover is inherently faster in SLE T cells. In contrast, ERα/ERβ mRNA and ERβ protein levels were not significantly different between SLE and normal control T cell samples. Plasma estradiol levels did not differ (*p* > 0.05) between SLE patients and controls. A previously undetected interaction between GR and ERα signaling pathways suggests posttranslational modification of steroid receptors in SLE T cells may alter ERα/GR actions and contribute to the strong gender bias of this autoimmune disorder.

## Introduction

Systemic lupus erythematosus (SLE) is a strongly gender-biased autoimmune disease affecting women nine times more frequently than men (1, 2). Onset and progression of SLE involves abnormal T cell signaling, stimulation of autoantibody production, and abnormal cytokine synthesis (3–6). The acquisition of SLE is linked to genetic susceptibility and diverse environmental agents can serve as triggers in genetically susceptible individuals to promote disease onset (4, 7, 8). However, the strongest risk factor for developing SLE is being female (1). The female sex steroid, estradiol, working through its receptors, contributes to the gender bias in SLE although the mechanisms are not well-understood (9–11).

Estradiol functions by binding to specific ERs, namely ERα and ERβ, which are members of a nuclear receptor ligand-regulated transcription factor family (12). Two independent genes that share a high degree of similarity in the DNA binding domain encode these receptors (13). In the classical mechanism of steroid hormone action, estradiol diffuses into target cells and binds to ERs in the nucleus (14). The ligand-activated receptors interact at specific DNA sites, termed estrogen response elements, along target genes and alter the rate of transcription (15, 16). Data from mice lacking ERα or ERβ suggest that each subtype performs specialized as well as overlapping functions to promote estradiol action *in vivo* (17). Male mice lacking functional ERα (ERα^−/−^) are resistant to developing a lupus phenotype in response to estradiol compared with their wild-type littermates suggesting ERα, rather than ERβ is responsible for inducing a lupus phenotype (18). This concept is supported by more recent data suggesting ERα promotes SLE in F1 females of a lupus mouse model (NZB × NZW) (19).

In female patients with SLE, T cell levels of ERα protein are lower after culture in estradiol, yet, T cells respond robustly to a ligand (ERα) selective agonist, 1, 3, 5-tris (4-hydroxyphenyl)-4-propyl-1H-pyrazole by stimulating calcineurin and CD154 mRNA expression (20). Genomic analysis of ER binding in breast cancer cell lines (21, 22) indicates a substantial overlap in the chromatin binding sites for ERα and ERβ when a single receptor form is expressed. However, less overlap occurs, and, a greater number of unique binding sites are occupied, when both receptor subtypes are expressed in the same cells (21). Both receptor subtypes are expressed in human T cells (20), and the possibility exists that the receptors could form functional heterodimers when co-expressed (23, 24).

Steroid receptors are regulated by a large number of posttranslational modifications including phosphorylation, acetylation sumoylation, and methylation (25–28). Conjugation of the small ubiquitin-like modifier SUMO (sumoylation) to acceptor lysine residues on substrate proteins occurs in a manner analogous to ubiquitination. Free SUMO is charged and transferred to an E2 ligase enzyme (UBC9), which acts in a catalytic manner to conjugate SUMO to an acceptor lysine. Once conjugated to SUMO, the substrate conformation changes with various functional consequences including alterations in protein-protein interactions, transcription, genomic stability and intracellular trafficking (28). Sumoylation and ubiquitin pathways are mechanistically similar but involve distinct enzymes and produce different cellular effects (28–31).

The hallmark of SLE is overproduction of autoantibodies that leads to irreversible, immune complex-mediated end-organ failure. Antibody responses depend on help from CD4^+^ T cells that are required for the generation of germinal centers where selection of high-affinity B cells and B cell memory occurs (32). Studies *in vitro* indicated that Th2 cells are the major T cell subset engaged in helping B cells (33). Subsequently, T cells expressing the chemokine receptor, CXCR5, were identified as the major T cell subset that provides help to B cells (34). These follicular helper T (Tfh) cells are recognized as a distinct Th subset (35–37). Tfh cells secrete a unique combination of effector molecules that are critical for their development and function including high levels of ICOS, CD154, and IL-21 that promote growth, differentiation, and class-switching of B cells (38, 39). Humans with impaired germinal-center formation through a deficiency of CD154 or ICOS have fewer CXCR5^+^ CD4^+^ T cells (40). Targeted deletion of CD154/CD40, ICOS or IL-21 and its receptor compromises the generation of robust germinal-center reactions and impairs humoral responses (39, 40). Involvement of Tfh cells in shaping the effector function of B cells, and in particular, the final differentiation step in plasma cells, implicates Tfh cells as key players in immune disorders such as SLE.

In SLE T cells, signal transduction pathways are altered by estradiol compared with normal T cells (41). Previous studies in our laboratory showed that estradiol could activate and repress genes within the same signal transduction pathway (41). Of particular interest was an increase in calcineurin and CD154 expression in SLE T cell samples but not in T cell samples from control females (9, 10). Upregulation of these genes in SLE T cells was expected to enhance calcium–calcineurin–NFAT signaling, ultimately resulting in exaggerated help to B cells and hypersecretion of autoantibodies. Consistent with this postulate was improved disease activity, and, a reduction in the expression of these T cell activation markers (calcineurin and CD154) in female SLE patients treated with Faslodex, a selective ERα antagonist (42).

The present study investigates changes in signal transduction pathways that could underlie a significant reduction in disease activity in SLE patients treated with Faslodex that we reported previously (42). The results suggest that estradiol, working through ERα, affects the expression of genes involved in Th cell differentiation. An unexpected interaction between ERα and GR signaling points to an intrinsic mechanism(s) in SLE T cells that alters receptor ubiquitination and sumolyation pathways. Changes in these pathways are expected to modify steroid receptor function, influence T cell development and may underlie the strong gender bias of this autoimmune disease.

## Materials and Methods

### Study Participants

This study was approved by the St. Luke’s Hospital Institutional Review Board and the Committee for the Protection of Human Research Subjects at Pittsburg State University. All subjects provided written informed consent prior to participation. Eighteen female patients who met the American College of Rheumatology criteria for classification of SLE (43) were enrolled in the study (Table 1). The patient’s systemic lupus erythematosus disease activity index (SLEDAI) ranged from mild to severe with a median SLEDAI value of 6 (range 2–18) at the time of blood draw. Ten of the patients were Caucasian, six were African American and two were of Asian descent. The age of the patients at the time of enrollment ranged from 24 to 51 years with a median age of 42 years. The SLE patients were taking various medications including azathioprine, mycophenolate mofetil, hydroxychloroquine, and prednisone (Pred) (Table 1). Twenty-five healthy control females were enrolled in the study. The control volunteers were between the ages of 21 and 48 with a median age of 46 years. Seventeen of the controls were Caucasian, four were African American, and four were of Asian descent. Participants had regular menstrual cycles and none of the patients or control females were taking oral contraceptives or exogenous hormone therapy at the time of blood draw. The patients and control volunteers had no history of other collagen vascular diseases.

**Table 1:** Systemic lupus erythematous (SLE) patient data.

Sample	SLEDAI	Medications	Disease duration (years)	Plasma estradiol (pg/ml)
SLE-1	4	MMF, Pret, HCQ	3	168.3
SLE-2	9	Pred	9	98.7
SLE-3	7	HCQ	5	90.3
SLE-4	2	Pred, HCQ	10	86.4
SLE-5	2	MMF, HCQ	12	128.7
SLE-6	4	Pred	3	84.5
SLE-7	12	MMF	11	98.4
SLE-8	5	Pred	32	123.7
SLE-9	4	HCQ, Pred	3	111.6
SLE-10	16	Pred, CycIo	22	ND
SLE-11	6	Pred, HCQ	6	ND
SLE-12	10	Pred	7	ND
SLE-13	6	Pred, HCQ	19	188.8
SLE-14	4	Pred	11	98.6
SLE-15	11	Pred, HCQ	20	ND
SLE-16	18	Pred, HCQ	15	ND
SLE-17	4	Pred	9	ND
SLE-18	6	Pred, HCQ, Aza	20	95.6

*Eighteen women with SLE volunteered for this study. The Systemic Lupus Erythematosus Disease Activity Index (SLEDAI) scores ranged between 2 and 18 at the time of enrollment. The patients were taking medications including mycophenolate mofetil (MMF), prednisone (Pred), hydroxychloroquin (HCQ), cyclophosamide (Cyclo), and azathioprine (Aza) as indicated. The duration of disease ranged from 3 to 32 years. Enrolled patients had regular menstrual cycles and were not taking exogenous hormones. Plasma estradiol was determined at the time of blood draw. ND, not determined*.

### Measurement of Plasma Estradiol

Plasma samples were isolated at the time of blood collection for T cells. Estradiol levels were measured by duplicate using a commercial ELISA plate (Estradiol ELISA 11-ESTHU-E01, ALPCO Diagnostics, Salem, NH, USA). A standard curve was used to determine the amount of estradiol in circulation at a wavelength of 450 nm. The intra-assay coefficient of variation was 5.85%.

### Collection of T Cell Enriched Peripheral Blood Mononuclear Cells

T cell enriched mononuclear cells were separated from blood samples (~90 ml) by density gradient (Histopaque, Sigma, St. Louis, MO, USA). Residual red blood cells were lysed (H-Lyse buffer, R&D Systems, Minneapolis, MN, USA). T cells were purified by negative selection through T cell isolation columns (Human T Cell Enrichment Columns, R&D Systems). The T cells were either used immediately (fresh T cell samples) or cultured overnight (18 h) at 37°C under 5% CO_2_ in serum-free medium (Hybridoma, Sigma, St. Louis, MO, USA) supplemented with l-glutamine (200 mM). Some T cells were activated after 18 h of culture for 4 h with phorbol 12 myristate 13-acetate (PMA, Sigma, 10 ng/ml) and ionomycin (Sigma, 0.5 µg/ml). Estradiol-17β (10^−7^ M) was added (or not) to half of the replicate cultures for the entire culture period. We have previously shown that this dose, which is at the upper physiological range of estradiol, effectively upregulates calcineurin and CD154 expression in SLE T cells as described in detail elsewhere (9, 10).

### T47D Cell Culture

T47D cells (ATCC, HTB-133, Manassas, VA, USA), a breast cancer cell line, which express ERα and ERβ were cultured at 37°C under 5% CO_2_ to 80% confluence (75 mm flask) in T47D media [RPMI (Cellgro, Manassas, VA, USA)] with 200 mM l-glutamine, 10% fetal bovine serum (Harlan Bioproducts, Madison, WI, USA), and penicillin (100 U/ml)-streptomycin (100 µg/ml) (Hyclone, Logan, UT, USA). The cells were released from the flask with trypsin-EDTA (Fisher Scientific, Fair Lawn, NJ, USA).

### RNA Isolation

RNA was isolated from T cells and T47D cells using the TRIzol reagent (Invitrogen, Carlsbad, CA, USA) and Phase Lock Gels Heavy (Eppendorf, Fisher Scientific). Total RNA was purified from T cells and treated with DNase I according to the manufacturer’s protocol (DNA-free, Ambion, Austin, TX, USA).

### Microarray Analysis

Gene profiling was carried out at the Kansas University School of Medicine Microarray Facility as described in detail elsewhere (41). The concentration and purity of total RNA was assessed with an Agilent Bioanalyzer and samples with RIN scores above 7.0 were used for complementary (cRNA) RNA synthesis. Biotinylated cRNA was hybridized to high density Affymetrix human GeneChips HG-U133_Plus_2, which contained 54,675 probe sets. The chips were scanned and analyzed using MAS5 type of data analysis with Affymetrix and Gene spring GX 7.3.1 (Agilent Technologies) software. Signal intensities of genes present in estradiol-treated activated T cell samples without and with Faslodex were compared to the non-treated activated T cell samples in order to generate a fold-change value. Differences greater than 1.5-fold were arbitrarily chosen for further study.

### Pathway Analysis

Cell signaling pathways were identified using the Ingenuity Pathways Analysis (IPA, Ingenuity Systems, Redwood City, CA, USA) library of canonical pathways. The canonical pathways are manually curated algorithms that transform gene lists into relevant signal transduction networks. Gene lists comprising the data sets from SLE patient’s T cells treated with and without estradiol and plus or minus Faslodex were entered into the IPA program and differences in gene expression among the treatments were matched to canonical pathways. Fischer’s exact test calculated a *p*-value that determined the probability that the association between the genes in the data set (treatment) and the canonical pathway (network) were explained by chance alone. The top canonical pathways are those with the largest number of gene matches within a signaling network.

### Real-time Polymerase Chain Amplification

Selected target genes within differentially regulated pathways were independently investigated by examining expression levels using real-time PCR. Total T cell RNA was digested using DNase 1 and cDNA was synthesized from 4 µg of the resulting RNA using a High Capacity cDNA kit (Applied Biosystems, Foster City, CA, USA). Real-time PCR (Step-one, Applied Biosystems) was carried out according to the manufacturer’s protocol. ERα and ERβ receptors were quantified from the same T cell template using a Taqman probe and ERα (Hs01046818, Applied Biosystems) and ERβ (Hs00230957, Applied Biosystems) primers. A Taqman probe and glyceraldehyde 3- phosphate dehydrogenase (Gapdh, Hs99999905, Applied Biosystems) specific gene primers were used for the internal control. The value of Ct was compared with a pooled T cell sample or T47D cell samples (ERα and ERβ) as positive controls. Samples without template were included in triplicate on each plate as a negative control. The relative expression levels were calculated by dividing the sample Ct values obtained from T cells cultured with and without estradiol from the same individual. The average Ct of Gapdh in untreated T cells was 21.8 ± SEM 1.8, whereas the average Ct of Gapdh in hormonally stimulated T cells was 22.3 ± SEM 1.5, indicating no change in response to treatment.

### Isolation of T Cell Proteins

Total RNA and proteins were sequentially separated from the same freshly isolated T cell samples by column purification (Norgen Biotek, ON, Canada). Briefly, RNA was bound to the column and the proteins were collected in the flow through. RNA was treated with DNase I and eluted from the column. The pH of the flow through was adjusted, the proteins were bound to the column, and the columns were washed. The proteins were eluted and stored at −80°C.

### Western Blot Analysis

Purified protein samples were heated at 95°C for 5 min. Total T cell proteins were size fractionated by sodium dodecyl sulfate polyacrylamide gel electrophoresis (SDS-PAGE, 10%). The T47D cell extract was used for a positive control for ERα and ERβ while the lysis solution served as a negative control. Proteins were transferred electrophoretically (18 h, 12 V) onto nitrocellulose membranes using Transblot buffer (25 mM Tris–HCL, pH 8.3, 192 mM glycine, and 20% methanol). After the protein transfer, non-specific protein binding sites were blocked with Superblock buffer (# 37515, Thermo Scientific, Rockford, IL, USA) for 1 h with gentle shaking. The membranes were incubated with an ERɑ rabbit polyclonal antibody (sc-542, Santa Cruz Biotechnology, Santa Cruz, CA, USA) in a 1:1,000 dilution for 1 h, and washed four times (5 min each) with wash buffer (1× PBS containing 0.05% Tween-20). The membrane was incubated with horseradish peroxidase-conjugated goat anti-rabbit IgG (10 µg/ml, 32460, Thermo Scientific, Rockford, IL, USA) at 1:4,000 dilution for 1 h. The blots were washed four times (5 min each) with wash buffer. The blot was incubated in a chemiluminescent reagent (Super Signal West Femto Maximum Sensitivity Substrate kit, 34096, Thermo Scientific, Rockford, IL, USA) for 5 min and exposed to chemiluminescent film (Kodak, BioMax) for 4–5 min. Blots were stripped with Restore Western Blot Stripping Buffer (Pierce, Rockford, IL, USA) for 15 min at 37°C to remove antibody. The membrane was exposed to chemiluminescent film for 5 min to ensure removal of the primary antibody. The membrane was reacted with ERβ antibody (sc-8974, Santa Cruz Biotechnology, Santa Cruz, CA, USA, 1:250 dilution) for 1 h. The membrane was incubated with horseradish peroxidase-conjugated goat anti-rabbit IgG (10 µg/ml, 32460, Thermo Scientific, Rockford, IL, USA, 1:4,000) for 1 h at 22°C with gentle shaking. The blot was incubated with Super Signal West Femto Maximum Sensitivity kit reagent for 5 min and the membrane was exposed to Biomax film for 4–5 min. The membrane was stripped and incubated with a β-actin antibody (2 mg/ml, A5441, Sigma, St. Louis, MO, USA, 1:6,000) for 1 h. The blots were washed and reacted with peroxidase-conjugated goat anti-mouse antibody (10 µg/ml, 32430, Thermo Scientific, Rockford, IL, USA) at a 1:4,000 dilution for 1 h. The blot was incubated with chemiluminescent substrate and exposed to Biomax film for approximately 10 s. The amount of receptor was determined using scanning densitometry (Kodak Gel Logic). The optical density of ERα and ERβ protein was divided by the optical density of β-actin on the same blot. Scanning densitometry of β-actin across assays did not vary more than 10% verifying its lack of response to treatment.

### Statistical Analysis

Samples from each subject enrolled in this study were not tested in all assays because the entire blood draw was required for each assay. Gene chips were normalized and a Student’s *t*-test was used to compare differences in T cell gene expression without estradiol and/or Faslodex and with estradiol and/or Faslodex. Comparison of differences in ERα/ERβ and CXCR5 expression were assessed using a nonparametric Mann–Whitney *U* test. A *p*-value < 0.05 (two-sided) was considered statistically significant.

## Results

Global changes in gene expression were compared between peripheral blood T cells of SLE patients (*n* = 9) cultured with and without estradiol in order to identify differential effects of estradiol on signaling pathways. The top five canonical pathways altered by estradiol treatment included Th cell differentiation, GR signaling, immune cell signaling in rheumatoid arthritis, cytokine communication between immune cells, and phospholipase C signaling (Table 2). The top five downstream genes altered by estradiol and shared among these canonical pathways are shown in Table 2. TNF and TGF-β1 were shared among four of the top five signaling pathways. IL-21 and NFATC3 were shared among two of the top five canonical pathways. Since the data were obtained from SLE T cells, we investigated if estradiol altered the canonical SLE signaling pathway. Estradiol changed gene expression in the SLE signaling pathway (p = 4.8 × 10^−3^), though this pathway was not among the top five significant pathways affected. Two candidate downstream genes, NFATC3 and TNF were shared between SLE and GR signaling pathways (Table 2).

**Table 2:** The top five canonical signal transduction pathways affected by estradiol in activated systemic lupus erythematosus (SLE) T cells.

Pathway	SLE + E vs SLE − E	Shared downstream genes (pathway)
1- T helper cell differentiation	2.7 × 10^−8^	TGF-β1 (1, 2, 3, 4)
2- Glucocorticoid Receptor Signaling	1.1 × 10^−7^	TNF (1, 2, 3, 4, 6)
3- Role of macrophages, fibroblasts, and, endothelial cells in rheumatoid arthritis	1.5 × 10^−^7	IL-21 (1, 3, 4) NFATC3 (2, 5, 6)
4- Role of cytokines in mediating commmnicalion between immune cells	1.9 × 10^−6^	RAF1 (2, 5)
5- Phospholipase C Signaling	3.2 × 10^−6^	
6- SLE signaling^a^	4.8 × 10^−3^	

*SLE T cell samples (*n* = 9) were cultured without and with estradiol. The T cells were activated as described in the text. Changes in global gene expression was profiled using microarray analysis. Gene lists were generated from the microarray data and pathways that were altered by estradiol were identified by Ingenuity Pathway Analysis. The *p*-value of overlap is shown for each pathway. Major downstream genes affected by estradiol treatment were variably shared among the pathways (shown in parentheses)*.

*^a^Although the canonical SLE signal transduction pathway was affected by estradiol it was not within the top five*.

**Figure 1:**
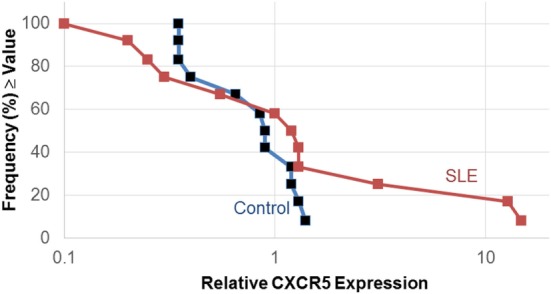
Estradiol effects on CXCR5 expression in activated systemic lupus erythematosus (SLE) T cells. Human T cells were isolated as described in the text and cultured in serum-free medium without and with estradiol. The relative amount of CXCR5 expression following activation was measured by real-time PCR. Data shown are the frequency of T cell samples from controls (*n* = 12) and SLE patients (*n* = 12) that exhibited relative CXCR5 expression values that were at or above the value on the *x*-axis.

The expression of the chemokine receptor, CXCR5 was different between SLE T cells treated without and with estradiol only in the Th cell differentiation pathway. Owing to the importance of CXCR5 in T–B cell interactions, we further quantified CXCR5 expression in SLE patient T cell samples (*n* = 12) cultured without and with estradiol using real-time PCR (Figure 1). In 3 of the 12 SLE T cell samples investigated, CXCR5 expression increased robustly (samples 5, 9, 16) in response to estradiol. The SLEDAI scores for those patients 2 (mild), 4 (moderate), and 18 (active) ranged from mild to severe. There was no correlation between disease activity and CXCR5 expression in the patients. The relative median expression of CXCR5 in SLE T cells was 1.1. In the control T cell samples (*n* = 12), CXCR5 expression did not change in response to estradiol (Figure 1). CXCR5 expression in the T cells from control females was generally unaffected by estradiol with a median relative expression of 0.9, similar to that for T cell samples from the SLE patients (Figure 1). Interestingly, relative CXCR5 expression varied in the SLE T cell samples with both lower and higher expression values compared to those from the control T cell samples.

Administration of the ERα antagonist Faslodex to SLE patients in a small clinical trial significantly reduced their SLEDAI scores (42). It was of interest, therefore, to identify signaling pathways that could account for disease improvement when ERα action was blocked by Faslodex. We compared SLE T cell samples (*n* = 9) cultured with Faslodex to the same T cell samples cultured without Faslodex (Table 3). In a separate set of experiments, we added estradiol to the SLE T cell cultures (*n* = 9) without and with Faslodex (Table 4). In the absence of added estradiol, the top canonical pathways affected by Faslodex are shown in Table 3. Addition of estradiol to the T cells cultured without and with Faslodex changed some of the top pathways affected (Table 4). It is notable, that GR signaling was a top canonical pathway affected by estradiol without and with added Faslodex (compare Tables 2–4). A striking relationship emerged for the top upstream regulators in SLE T cells treated with Faslodex, regardless of estradiol addition or not. The principal regulators shared among Faslodex treated T cells included MYC, HNF4A, ESR1, and CST5 (Tables 3 and 4). Downstream genes affected by ERα antagonism and shared among the Faslodex treatments, regardless of added estradiol, included AKT3, CBL, cyclic AMP responsive element modulator (CREM), FOS, JUN, and NFAT5. In the absence of added estradiol, MMP3 was a unique upstream regulator (Table 3), while addition of estradiol to cultures revealed TP53 as a unique regulator (Table 4). The SLE canonical signaling pathway was significantly altered in SLE T cells cultured in Faslodex containing medium without (7.7 × 10^−4^) and with (3.4 × 10^−8^) estradiol, though it was not one of the top five canonical pathways.

**Table 3:** The top five canonical pathways affected by the estrogen receptor-alpha (ERα) antagonist, Faslodex, in activated systemic lupus erythematosus (SLE) T cells.

Pathway	SLE-E + F vs SLE-E	Tap upstream regulators
Glucocorticoid receptor signaling	2.8 × 10^−6^	HNF4A
Sumoylation pathway	2.2 × 10^−5^	MVC
Purine nucleotides biosynthesis II	8.6 × 10^−5^	ESR1
Estrogen receptor signaling	1.1 × 10^−4^	CSTS5
Cleavage and polyadenyladon of Pre-mRNA	1.8 × 10^−4^	MMP3
SLE signaling^a^	7.7 × 10^−4^	

*SLE T cell samples (*n* = 9) were cultured without and with Faslodex and activated as described in the text. Gene lists were generated from microarray data and analyzed for canonical signaling pathways altered by Faslodex. The *p*-value of overlap is shown for each pathway*.

*^a^Although the canonical SLE signal transduction pathway was affected by Faslodex, it was not within the top five. MMP3 was a unique top upstream regulator in the T cells treated with Faslodex without estradiol addition*.

**Table 4:** The top five canonical pathways affected by the estrogen receptor-alpha (ERα) antagonist, Faslodex in activated systemic lupus erythematosus (SLE) T cells (*n* = 9) cultured with estradiol was determined.

Pathway	SLE + E + F*vs* SLE + E	Top Upstream Regulators
Glucocorticoid receptor signaling	1.1 × 10^−16^	MYC
EIF2 signaling	3.8 × 10^−15^	HNF4A
Hereditary breast cancer signaling	1.6 × 10^−11^	ESR1
Protein ubiquitination pathway	9.6 × 10^−10^	CSTS5
JAK/Stat Signaling	1.4 × 10^−9^	TP53
SLE signaling^a^	3.4 × 10^−8^	

*Gene lists were generated from microarray data and analyzed for canonical signaling pathways altered by Faslodex. The p-value of overlap is shown for each pathway*.

*^a^Although the canonical SLE signal transduction pathway was affected by Faslodex plus estradiol, it was not within the top five. TP53 was a unique top upstream regulator in the T cell samples treated with Faslodex with estradiol addition*.

ER antagonism altered the ubiquitination (SLE T cells + estradiol + Faslodex vs SLE T cells + estradiol) pathway in SLE T cells. This pathway was of particular interest because ubiquitin enzymes are essential for regulation of T cell, B cell, and TNF signaling cascades (44). Moreover, activated SLE T cells cultured in medium containing estradiol express less ERα protein than T cell samples from healthy women cultured under the same conditions (20). Investigation of the downstream genes that differed between SLE T cells cultured with estradiol and treated without and with Faslodex revealed changes in 57/255 genes in the ubiquitination pathway (data not shown). The affected genes included ubiquitin-activating enzymes (E1), conjugating enzymes (E2), ligases (E3 HECT), and deubiquitinases. Several factors within the immunoproteosome were altered and the transporter associated with antigen processing (TAP) differed in SLE T cells treated with Faslodex compared with untreated SLE T cells.

ERα antagonism altered sumolyation (SLE T cells − estradiol + Faslodex vs SLE T cells − estradiol) signaling in SLE T cells. Within the sumoylation pathway, the expression of 27/96 genes were affected by Faslodex treatment. Investigation of the genes involved, revealed expression of SUMO-1, FAS, RANBP2 and GR as candidates modified by Faslodex treatment. Since Faslodex altered GR signaling in all SLE T cells, we also compared the data sets to determine which key downstream genes were altered. Faslodex changed the expression of 62 genes (62/282) in the absence of added estradiol and 76 genes (76/287) when estradiol was added to the activated SLE T cell cultures (data not shown). Changes in key downstream genes revealed differences in SUMO-1 and UBE21 (UBC9) expression (data not shown). SUMO-1 and UBC9 target nuclear hormone receptors and their ability to modulate transcription.

To test if protein turnover of ERα was modified in SLE T cells, we compared mRNA and protein levels in freshly isolated T cells from SLE patients and control females. Receptor mRNA and protein were quantified in nine freshly isolated SLE T cell samples (Table 5) and 10 freshly isolated control T cell samples (Table 6). Comparison of the amount of ERα subtype mRNA revealed no significant differences (*p* = 0.97) between SLE patient and control T cell samples. The median relative value for ERα mRNA in SLE T cell samples was 0.027 (Table 5) while the median relative value in control T cell samples was 0.045 (Table 6). Comparison of the amount of ERβ subtype mRNA revealed no significant differences (*p* = 0.18) between SLE patient and control T cell samples. The median relative value for ERβ mRNA in the SLE T cell samples was 0.6 (Table 5) while the median value in the control T cell samples was 2.5 (Table 6). The difference in the ratio of ERα: ERβ mRNA between the control and SLE T cell samples approached significance (*p* = 0.065), even though the ratio was less than unity for all samples in both groups. The median concentration of estradiol in plasma was similar (*p* = 0.28) between the SLE patients (97 pg/ml, Table 5) and the control females (123 pg/ml, Table 6). Those values are within the normal range for women with regular menstrual cycles.

**Table 5:** Measurements of ER subtype mRNA and protein in freshly isolated systemic lupus erythematosus (SLE) T cells.

Samples	ERɑ	ERβ	ERɑ	ERβ	Estradiol pg/ml
	
	(mRNA)		(Protein)		
SLE-1	0.036	0.811	0.62	0.61	166.3
SLE-2	0 026	0.170	0.35	0.85	98.7
SLE-3	0.018	0.443	0.93	0.80	90.3
SLE-4	0.027	0.442	0.29	0.67	86.4
SLE-5	0.083	1.365	0.40	0.87	128.7
SLE-6	0.071	0.370	1.00	0.90	84.5
SLE-7	0.015	0.660	0.31	0.65	98.4
SLE-8	0 024	0.600	0.80	0.98	123.7
SLE-9	0.238	18.15	0.76	0.98	111.6

Median	0.027	0.60	0.62	0.85	98.7

*Blood samples were drawn from SLE patients (*n* = 9) and T cells were purified by negative selection. ER subtype mRNA was measured by real-time PCR and protein in the same T cell sample was quantified by Western blotting. Plasma estradiol was measured at the time of blood draw. The levels of hormone in circulation were within the normal range for women with regular menstrual cycles*.

*SLEDAI, systemic lupus disease activity index*.

**Table 6:** Measurements of ER subtype mRNA and protein in freshly isolated systemic lupus erythematosus T cells.

Samples	ERɑ	ERβ	ERɑ	ERβ	Estradiol(pg/ml)
	
	(mRNA)		(Protein)		
CTRL 1	0.068	7.40	1.10	0.88	99.5
CTRL 2	0.020	0.63	1.00	0.92	156.5
CTRL 3	0.004	0.08	1.60	1.60	88.9
CTRL 4	0.030	2.70	0.90	1.20	133.3
CTRL 5	0.008	0.30	1.10	1.40	99.6
CTRL 6	0.009	3.70	0.67	0.08	124.8
CTRL 7	0.060	4.70	0.83	1.20	143.9
CTRL 8	0.147	1.80	0.81	0.97	125.7
CTRL 9	0.090	2.20	0.63	0.46	121.5
CTRL 10	0.270	7.50	1.10	1.00	93.3

Median	0.045	2.5	0.97	0.99	123.1

*Blood samples were drawn from healthy volunteers (*n* = 10) and T cells were purified by negative selection. ER subtype mRNA was measured by real-time PCR and protein in the same T cell sample was quantified by western blotting. Plasma estradiol was measured at the time of blood draw. The levels of hormone in circulation were within the normal range for women with regular menstrual cycles*.

*SLEDAI, systemic lupus disease activity index*.

**Figure 2:**
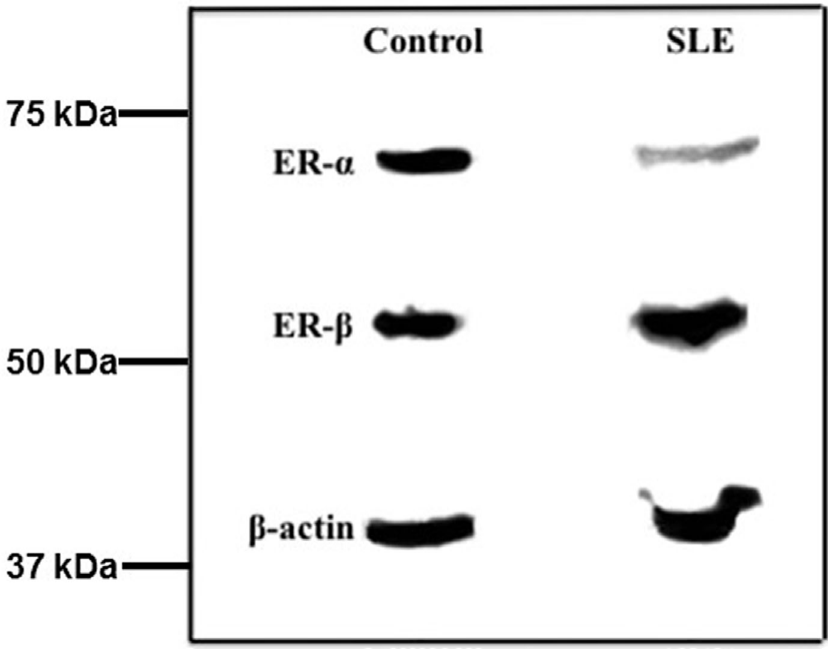
Western blots of freshly isolated T cell proteins indicate the amount of ERα but not ERβ is less in systemic lupus erythematosus (SLE) patients compared with female controls. Fresh T cell extracts were size fractionated by SDS-PAGE and transferred to nitrocellulose membranes. The blots were sequentially reacted with antibodies to ERα, ERβ, and β-actin. The relative amount of receptor subtype was measured by scanning densitometry and values were adjusted to β-actin in the same T cell sample.

We next analyzed ER subtype protein expression in the same T cell samples. Incubation of western blots with ERα antibody revealed a single reactive protein that migrated at approximately 65 kDa, consistent with the size for ERα protein (Figure 2). After the membrane was stripped and reacted with ERβ antibody a single reactive protein was identified at approximately 56 kDa, consistent with the size of ERβ (Figure 2). The membrane was stripped and reacted with β-actin antibody and a single reactive protein was observed at a molecular size of approximately 42 kDa, consistent with the size for β-actin. In the absence of T cell extract, no reactive proteins were observed (data not shown).

Comparison of the amount of ERα protein revealed a significant difference (*p* = 0.010) between SLE patient and control T cell samples. The median relative value in SLE T cell samples was 0.62 (Table 5) while that for ERα protein in control T cell samples was 0.97 (Table 6). There was no significant difference in the median values for ERα between patients with mild disease (median value 0.56, SLEDAI ≤ 4) and those with greater disease activity (median value 0.61, SLEDAI ≥ 5). Comparison of the amount of ERβ subtype protein revealed no significant differences (*p* = 0.11) between SLE patient and control T cell samples. The median relative value in SLE T cell samples was 0.85 (Table 5) while the median relative value for ERβ protein in control T cell samples was 0.99 (Table 6). The ratio of ERα: ERβ protein was a median of 0.78 in the SLE T cell samples and 1.05 in the control T cell samples, respectively. The difference in the ratio of ERα: ERβ protein between the SLE and the control T cell samples was not significant (*p* = 0.079).

Comparison of the ratio of protein to mRNA (designated as the relative productivity) showed all ratios > 1 for ERα while ratios for ERβ were < 1 in the majority (11/19) of samples. The relative productivity of ERα was always greater than for ERβ, but these did not differ between the two cohorts (Table 7). However, a comparison of the relative subtype expression (ERα:ERβ) for the protein:mRNA ratios between the SLE T cell samples and the normal T cell samples revealed lower values in the SLE T cell samples (*p* = 0.018, Table 7). The primary factor associated with ERα protein was ERβ protein (*p* = 0.006). The second factor associated with ERα protein was experimental group (cohort, i.e., SLE T cell samples vs control T cell samples).

**Table 7:** Comparison of the relative expression of estrogen receptor subtypes (ERα:ERβ) for protein:mRNA ratios indicates lower values in the systemic lupus erythematosus (SLE) T cell samples.

Parameter	Medlar control	Values SLE	*p*-Value (Mann–Whitney)
Ratio of protein:mRNA (Erα)	23	14	0.37
Ratio of protein:mRNA (ERβ)	0.3	1.5	0.14
Ratio of protein:mRNA (ERa:ERp)	34	21	0.018

*These results suggest accelerated turnover of ERα in SLE T cells*.

## Discussion

The present study investigated global cell signaling changes in human SLE T cells treated with estradiol and the ERα antagonist, Faslodex. We compared the effects of blockading the action of ERα in order to identify signaling pathways that could contribute to improved disease activity in women with SLE we reported previously (42). Estradiol altered gene expression in pathways involved in Th cell differentiation, ERα/GR signaling and immune cell interactions. Antagonism of ERα by Faslodex revealed changes in protein ubiquitination and protein sumoylation pathways. We found that ERα protein but not mRNA was lower in SLE T cells compared with T cells from healthy individuals, suggesting more rapid turnover of ERα in SLE T cells. The results are consistent with the concept that turnover of ERα is accelerated in SLE T cells and may occur through alterations in the ubiquitination signaling pathway. Antagonism of ERα affected the sumoylation pathway and SUMO-1 and UBC9 expression were changed in GR signaling. Modification in these signaling pathways could account for the significant improvement of disease activity in SLE patients receiving monthly Faslodex treatments (42). Additional experiments are required to determine how changes of steroid receptors could alter signal transduction pathways in SLE T cells as suggested by the current results.

ERα protein levels are lower in SLE T cells compared with normal T cells although ERα mRNA and ERβ mRNA and protein are similar between SLE T cell samples and control T cell samples. The molecular basis for the difference in ERα protein levels remains to be established but the current results suggest ERα turnover accelerates owing to changes in the ubiquitination pathway. The half-life of ERα protein is short (~4 h) in primary uterine cells and breast cancer cell lines in culture (45). Consistent with other short-lived regulatory proteins, ERα turnover occurs through the 26S proteasome system (46–48). In spite of the shared structural similarities between the receptors, our results suggest that ERα and not ERβ is the target for accelerated turnover. A recent study in breast cancer cell lines revealed the S-phase kinase-associated protein 2 (*Skp2*), which is a substrate recognition component of the SC ubiquitin ligase complex, targets ERα but not ERβ for degradation (49). The basis of this difference resides in a serine residue (serine 294), which is phosphorylated in ERα by p38MAPK and is not present in ERβ. In SLE T cells treated with Faslodex, MAPK expression was altered in the ERα signaling pathway (data not shown). However, patterns of ERα phosphorylation, activation and turnover require additional analyses in T cells. Since appropriately timed destruction of ERα is essential for its function, it is now important to investigate potential posttranslational modifications of ERα that may accelerate receptor turnover in SLE T cells and change the transcriptional activity of the receptor.

SUMO modification of ERα and GR alters protein–protein interactions and transcriptional profiles (25, 28, 50). The enzyme, UBC9, is the only known E2 SUMO-conjugating enzyme that is necessary for SUMO attachment to substrate proteins (28, 30, 31). Comparison of SLE T cells cultured with estradiol and Faslodex to the same T cell sample cultured with estradiol alone (Table 4), revealed changes in GR signaling. Downstream genes affected in the GR canonical signaling pathway included *Sumo1* and *Ubc9*. Estradiol increased UBC9 expression in MCF-7 breast cancer cells while ICI182,780 abrogated the response (51, 52). *Ubc9* deletion in T regulatory (Treg) cells results in early-onset lethal autoimmune disorders (53). Loss of *Ubc9* downregulated a variety of cytokine, chemokine and IL-1 receptor activity suggesting that sumoylation is required for proper immune function of Treg cells. Mice deficient in *Ubc9* show perturbations in early T cell maturation in the thymus and a reduction in the nuclear localization of NFAT in response to PMA-ionomycin activation (54). The results from our study suggest ERα antagonism changes the expression of genes involved in GR signal transduction in SLE T cells. To our knowledge, interaction between ER and GR signaling has not been studied in SLE T cells. However, in murine mammary cells chromatin accessibility is enhanced by activation of the opposite receptor (55), GR activation can displace ER from AP-1 sites (56), and ER and GR may act cooperatively at DNA regulatory sites (57). Disruption of GR signaling results in inflammation characterized by increased cytokines in the blood (58). The results from our study suggest that interaction between ERα and GR occurs in SLE T cells when ERα action is blocked. Increased understanding about the molecular basis of this interaction could explain the improvement in SLE patient’s disease activity when Faslodex was administered monthly to SLE patients.

Antibody responses depend on help from CD4^+^ T cells that are required for the generation of germinal centers where selection of high-affinity B cells and B cell memory occurs (32). Expression of CXCR5, when coupled with loss of the T cell zone-homing chemokine receptor CCR7, allows Tfh cells to relocate from the T cell zone to the B cell follicles, where they support B cell expansion and differentiation (59). In the present study, estradiol increased CXCR5 expression in 25% of the T cells from SLE patients. The difference in expression was primarily due to three T cell samples in which, expression levels robustly responded to estradiol. We did not find a correlation between CXCR5 expression and SLEDAI scores, but our study measured relative expression rather than the number of CRCR5^+^ cells. Tfh cells in circulation constitute a small subset of total immune cells in the blood. Thus, it is possible that the median change in CXCR5 expression in circulating T cells is due to an increase in the number of cells expressing CXCR5 but additional experiments are necessary to resolve this question. Dysregulation of Tfh cells that promotes B cell activation is associated with SLE-like disease in the roquin san/san mouse (60, 61). This mouse model arose from a mutation in the ubiquitin ligase roquin that disrupts a repressor of ICOS, an essential stimulator of Tfh cells. Analysis of CXCR5^+^ CD4^+^ cells expressing high levels of Tfh-associated molecules, revealed a subset of SLE patients who showed increased Tfh cells in circulation. The increased Tfh cells correlated with the diversity and titers of autoantibodies and with the severity of end-organ involvement (62). Analysis of global gene expression in this study, indicate Th cell differentiation is affected by ERα antagonism. Since CXCR5 is a defining marker for Tfh cells, we explored changes in CXCR5 expression in SLE T cells. The results are equivocal because only 3 out of 12 SLE T cell samples were estradiol responsive. Moreover, Th responses can be mediated by Th1/Th2 and Th17 subsets (35, 37, 63). In order to define the role of ERα in Th differentiation, analysis of Th subsets and downstream effector functions are necessary.

Systemic lupus erythematosus is a multifactorial autoimmune disorder with numerous cellular abnormalities and clinical presentations. The unifying theme among SLE T cell dysfunction is a loss of the ability to sense antigenic signals and properly integrate these signals within the adaptive and innate immune systems. While progress has been made in understanding the molecular basis and genetic susceptibility for SLE, the strong gender bias in the disorder remains an enigma. The present study indicates a significant decline in the amount of ERα protein in resting SLE T cells relative to resting normal T cells. Alterations in the balance of ERα and ERβ will profoundly affect hormone-responsiveness of target cells as suggested from global analyses of ER subtype binding across the genome. Analysis of global changes in gene expression when we blocked ERα function with Faslodex in SLE T cells indicates both protein ubiquitination and sumoylation pathways are affected. Faslodex identified an unsuspected interaction between ERα and GR signaling. Steroid receptor function requires appropriately time destruction and sumoylation of receptors. The present results suggest that posttranslational modification of steroid receptors (ERα/GR) in SLE T cells may be aberrant. These alterations are likely to affect numerous pathways and lead to signaling dysfunction of SLE T cells.

In this study, we have focused on female SLE patients with regular menstrual cycles who were not taking exogenous estradiol. Although the study design controlled for exogenous estradiol effects, SLE is a heterogeneous autoimmune disorder with numerous clinical presentations. Current therapeutic agents used to treat SLE are based on patterns of end-organ appearance rather than from understanding the molecular basis of the disease. Since the goal of this study was to assess the importance of estradiol in SLE T cells, we did not select a homogeneous group of patients to study based on end-organ involvement. However, none of the patients, at the time of study, had active renal or central nervous system disease. Patients presented with polyarticular non-erosive arthritis (*n* = 16), proteinuria with normal renal function (*n* = 7), pleuritis (4), and lupus malar rash or discoid lupus rash (*n* = 7). Because the study population was heterogeneous, we cannot conclude that the results are representative of all SLE patients. Moreover, it is important to consider that immunosuppressant drugs, such as Pred, may have affected the results. Additional studies including SLE patients not taking medications are necessary to clarify this issue. Although SLE is a strongly gender-biased disorder, the disease occurs in males. The features of SLE in males is often more severe than in female patients (4). Future studies should include male SLE patients to determine if steroid receptor turnover is modified in male patients or accelerated turnover is gender specific. It will be interesting to investigate the interaction of steroid receptors, including the androgen receptor, with various cofactors that may alter gene expression and lead to the onset or progression of SLE. A recent study in a breast cancer cell line reported that GR represses ERα action (64). The repression of ERα-dependent transcription appeared to be contingent on GR sumoylation, which caused the recruitment of GR and a corepressor complex to ERα occupied enhancers. Greater understanding of how posttranslational modifications of steroid receptors integrate immune-endocrine signaling will help in the molecular understanding of gender-biased autoimmunity. Ultimately, this knowledge will permit greater precision in diagnosis and treatment of patients with SLE and lead to better patient outcomes.

### Datasets Are in a Publicly Accessible Repository

The datasets generated and analyzed in this study can be found on the KUMC public repository: (http://bioinformatics.kumc.edu/mdms/shares/data/VRider_exp1_raw_leixmWnf1mGhM/).

## Author Contributions

VR prepared the T cells, conducted the experiments and IPA analysis, and wrote the manuscript. NA enrolled the SLE patients, collected the blood samples, and provided patient data. BK carried out the statistical analyses and contributed to data interpretation. NL, SB, and BF generated the gene lists from the microarray data.

## Conflict of Interest Statement

The authors declare that there is no conflict of interest that could be perceived as prejudicing the impartiality of the research reported.
